# Synthesis of a mitochondria-targeted spin trap using a novel Parham-type cyclization

**DOI:** 10.1016/j.tet.2009.07.081

**Published:** 2009-09-26

**Authors:** Caroline Quin, Jan Trnka, Alison Hay, Michael P. Murphy, Richard C. Hartley

**Affiliations:** a Centre for the Chemical Research of Ageing, WestCHEM Department of Chemistry, University of Glasgow, Glasgow, G12 8QQ, UK; b MRC Mitochondrial Biology Unit, Hills Road, Cambridge, CB2 0XY, UK

## Abstract

A new cyclic nitrone spin trap, [4-(3′,3′-dibutyl-2′-oxy-3′*H*-isoindol-5′-yloxy)butyl]triphenylphosphonium bromide (MitoSpin), bearing a lipophilic cation has been prepared by a route that involves a novel Parham-type lithiation–cyclization of an isocyanate to give the isoindolinone core. MitoSpin accumulates in a membrane potential dependent way in energized mitochondria and its oxidation could potentially be used in the study of oxidative stress resulting from reactive oxygen species generated in mitochondria.

## Graphical abstract

**Figure:**
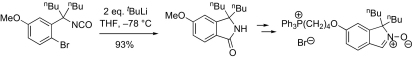

## 1 Introduction

**Scheme 1:**
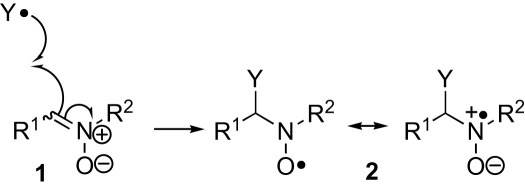
Spin-trapping with nitrones.

Mitochondria play a central role in energy metabolism and cell death, and consequently mitochondrial dysfunction contributes to many pathologies.^1^ It has been suggested that reduction of oxygen to superoxide by the mitochondrial respiratory chain ultimately leads to the production of highly reactive oxygen-centered radicals and carbon-centered radicals, which are then responsible for many of these pathologies and for aging on a cellular and whole organism level.^2,3^ Nitrones **1** react with these highly reactive oxygen-centered and carbon-centered radicals (Y^•^) to give nitroxides **2** that are more stable and longer lived (Scheme 1).^4^ The nitroxides **2** can be detected by EPR spectroscopy and the hyperfine splittings observed can often be used to identify the radical that led to their formation. Thus, nitrones can be used as so-called spin traps for the study of radical processes in biological samples and there is interest in making spin traps that would accumulate within mitochondria and report on mitochondrial radical production.^5–8^

**Figure 1:**
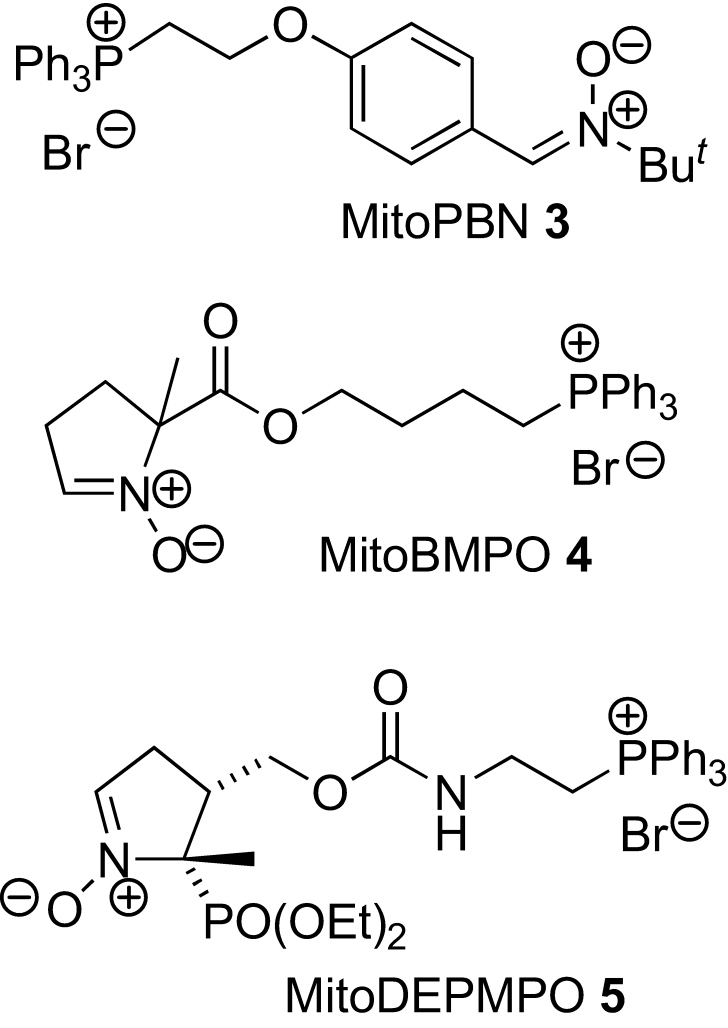
Mitochondria-targeted spin traps.

Small molecules can be targeted to mitochondria by conjugation to lipophilic cations such as the alkyltriphenylphosphonium (TPP) cation.^9,10^ These lipophilic cations easily permeate biological membranes due to their hydrophobicity and large ionic radius, and the large mitochondrial membrane potential (150–170 mV, negative inside) causes the several-hundred fold accumulation of these lipophilic cations into the mitochondrial matrix in accordance with the Nernst equation.^9–11^ This approach has been used to target antioxidants to mitochondria in cells, in vivo and in patients as a therapeutic approach^10,12,13^ and to drive the accumulation of probe molecules within mitochondria.^14–16^ Recently, three different spin traps have been targeted to mitochondria by conjugating them to TPP cations: α-phenyl-*N*-*tert*-butylnitrone (PBN),^5^ 5-*tert*-butoxycarbonyl-5-methyl-1-pyrroline *N*-oxide (BMPO),^8^ and 5-(diethoxyphosphoryl)-5-methyl-1-pyrroline *N*-oxide (DEPMPO)^6,7^ (Fig. 1). The PBN derivative **3** produces long-lived nitroxide radicals with carbon-centered radicals, but not with oxygen-centered radicals, while the targeted BMPO and DEPMPO spin traps **4** and **5** also react with oxygen-centered radicals to generate relatively stable spin adducts, which can be identified by their hyperfine couplings.

**Scheme 2:**
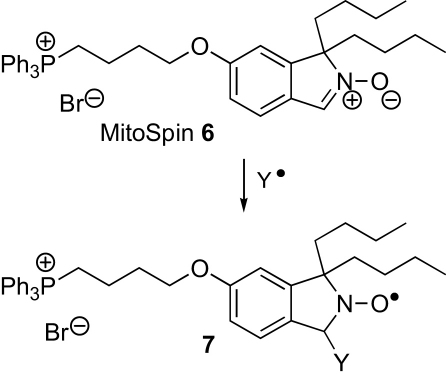
Intended spin-trapping of radicals (Y^•^) by MitoSpin **6**.

One problem with nitroxides is that they are rapidly converted into EPR-silent products in vivo through their reduction by ascorbate, glutathione, and ubiquinol,^17^ and there is interest in developing spin traps that generate adducts that are resistant to such reduction. 1,1,3,3-Tetraalkylisoindolin-2-yloxyl radicals^18,19^ are reduced more slowly than many,^18^ and we designed MitoSpin **6** as a spin trap that would be targeted to mitochondria by a TPP cation and was expected to generate sterically protected (albeit less so than the tetralkyl derivatives) isoindolin-2-yloxyl radicals **7** by reaction with highly reactive oxygen-centered and carbon-centered radicals (Y^•^) (Scheme 2). The nitrone moiety would be conjugated with an electron-donating alkoxy group to enhance reactivity with electron-deficient oxygen-centered radicals, and since the benzylic C–N bond of spin adducts **7** would be held in a ring perpendicular to the π system, this conjugation should not encourage the fragmentation of the spin adducts. The alkyl groups would serve the dual role of encouraging interaction with the mitochondrial inner membrane (the site of the main source of free radicals within mitochondria) and sterically protecting the radical center in the adduct, while the presence of two tails rather than one should also discourage micelle formation.^20^

## 2 Results and discussion

**Scheme 3:**
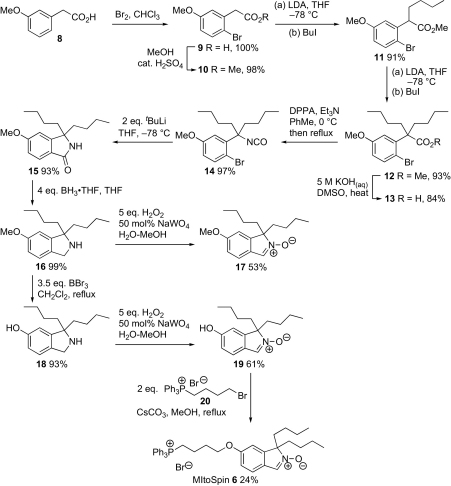
Synthesis of MitoSpin **6**.

Fevig and co-workers had prepared a nitrone related to MitoSpin **6**, via Lewis-acid mediated cyclization of an isocyanate to give an isoindolinone core.^21^ However, in preliminary studies, we found a similar cyclization unsatisfactory, so MitoSpin **6** was prepared by first brominating 3-methoxyphenylacetic acid **8** to give acid **9**. This was converted into ester **10** and two consecutive enolate alkylations produced ester **12** via ester **11**. Conversion to the free acid **13** could be achieved in base or by S_N_2 reaction with lithium iodide, and reaction with diphenylphosphoryl azide (DPPA) followed immediately by Curtius rearrangement of the acyl azide gave isocyanate **14**. Rather than hydrolyzing the isocyanate **14** to give an amine for transition metal-mediated carbonylation with carbon monoxide,^22^ we decided to investigate whether a Parham-type procedure^23^ involving lithium–bromine exchange followed by intramolecular reaction between the resulting aryllithium and the isocyanate would give amide **15** directly. Couture and co-workers had shown that isoindolinones could be made from carbamates in this way,^24^ and intermolecular reaction between phenyllithium and an isocyanate to produce an acyclic amide was known,^25^ but the intramolecular version of this reaction was new. Therefore, we were delighted to find that the isocyanate **14** gave amide **15** in high yield when treated with *tert*-butyllithium. Reduction to give amine **16** proceeded smoothly. Oxidation of amine **16** to nitrone **17** followed by deprotection to give phenol **19** was successful, but reversing the steps to give the phenol **18** first was more efficient. Finally, coupling with commercially available phosphonium salt **20** gave MitoSpin **6**, albeit in modest yield (Scheme 3).

**Figure 2:**
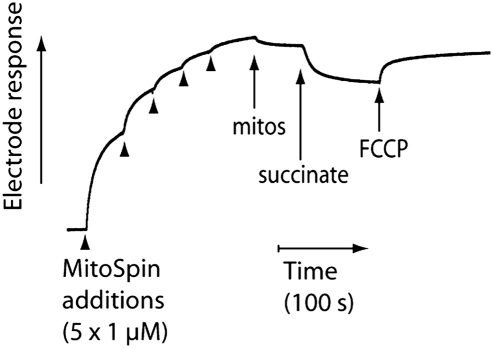
Uptake of MitoSpin by energized mitochondria. An electrode sensitive to the TPP moiety of MitoSpin was inserted into 3 mL KCl buffer supplemented with 4 μg/mL rotenone with stirring at 37 °C and five consecutive 1 μM additions of MitoSpin were used to calibrate the response of the ion-selective electrode (arrowheads). The addition of rat liver mitochondria (mitos; 1 mg protein/mL), succinate (10 mM), and FCCP (1 μM) are indicated. This result is typical of three independent measurements.

To assess whether MitoSpin **6** accumulated within mitochondria driven by the membrane potential we used an ion-selective electrode that responds to the triphenylphosphonium (TPP) moiety (Fig. 2). The electrode was inserted into a stirred, thermostated incubation chamber and showed the expected logarithmic response to five sequential additions each increasing the concentration of MitoSpin **6** by 1 μM to give a final concentration of 5 μM (Fig. 2, arrowheads show additions). Isolated mitochondria were then added in the presence of the respiratory inhibitor rotenone to prevent generation of a membrane potential from endogenous substrates. This led to a slight decrease in the concentration of MitoSpin due to the expected adsorption of the compound to the mitochondrial surface.^26,27^ Addition of the respiratory substrate succinate to generate a mitochondrial membrane potential led to a rapid decrease in the concentration of MitoSpin **6**, consistent with its uptake into the mitochondrial matrix on induction of a membrane potential. To confirm that the uptake was due to the mitochondrial membrane potential, we next added the uncoupler carbonylcyanide *p*-(trifluoromethoxy)phenylhydrazone (FCCP), which abolished the mitochondrial membrane potential and led to a rapid efflux of MitoSpin **6** from the mitochondria. The electrode response to hydrophobic TPP compound is semi-quantitative due to electrode drift.^26^ Even so, we can estimate that under these conditions the steady state concentration of MitoSpin **6** outside energized mitochondria is approximately 3 μM and that the remainder of this is accumulated inside the mitochondria. From the incubation chamber volume and the intramitochondrial volume (∼0.6 μL/mg protein^28^) this corresponds to approximately 3 mM concentration of MitoSpin **6** inside mitochondria, consistent with the ∼1000-fold accumulation by the mitochondria expected from the Nernst equation for mitochondria with the expected mitochondrial membrane potential values of 150–170 mV.^9^ Therefore, these data are consistent with uptake of MitoSpin **6** into mitochondria driven by the mitochondrial membrane potential, indicating that MitoSpin **6** behaves in a similar way to other hydrophobic TPP-conjugated compounds and that on addition to mitochondria it is rapidly taken up into the matrix.^29^

**Figure 3:**
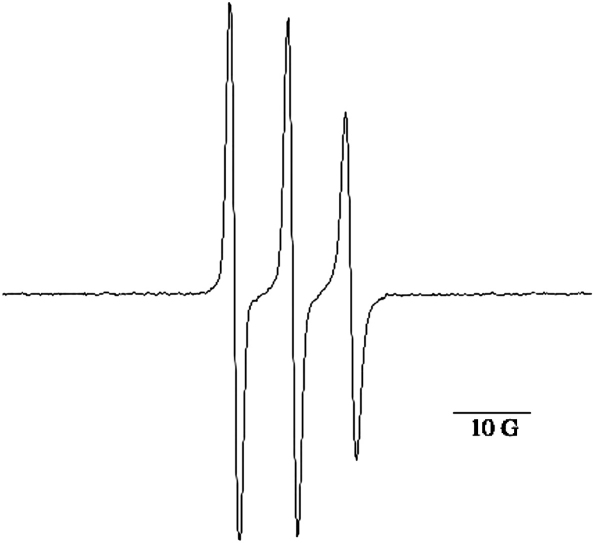
EPR spectrum of MitoSpinox **23**, *g*=2.0071, *A*_N_=7.6 G, the bar indicates the scale for the magnetic field strength in Gauss.

**Scheme 4:**
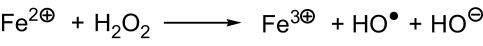


**Scheme 5:**
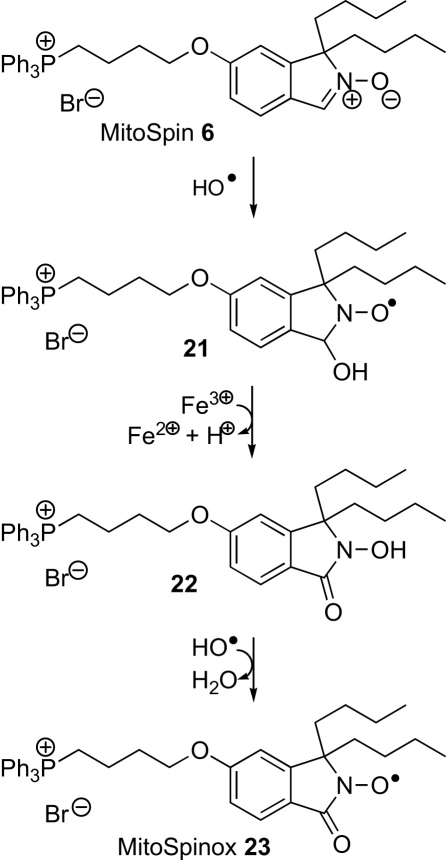


Next MitoSpin **6** was assessed as a spin trap. The Fenton reaction between iron(II) ions and hydrogen peroxide was used to generate hydroxyl radicals (Scheme 4) in the presence of MitoSpin **6**, and the EPR spectrum obtained at a range of concentrations of each reactant was a narrow triplet (Fig. 3). The same spectrum was obtained when MitoSpin and hydrogen peroxide were irradiated with UV light. The signal was very stable (72 h, without significant change), but could be abolished by treatment of the solution with glutathione or ascorbic acid. This narrow triplet is not consistent with the hydroxy adduct **21** and is assigned as MitoSpinox **23** (Scheme 5), which has no β-hydrogen atom. A related nitroxide, 5,5-dimethyl-2-pyrrolidone-1-oxyl (DMPOX), has a similar small hyperfine splitting of *A*_N_=7.1 G for coupling with the nitrogen nucleus.^30^ Under the Fenton conditions it seems likely that the originally generated hydroxy adduct **21** is oxidized to the EPR-silent hydroxamic acid **22** (represented as the more thermodynamically stable tautomer^31^) by reaction with iron(III) ions. A similar reaction has been reported for DMPO.^32^ Hydroxamic acid **22** could also be formed by disproportionation of nitroxide **21** or by abstraction of a hydrogen atom from the nitroxide **21** by a second hydroxyl radical. Reaction between hydroxamic acid **22** and a hydroxyl radical would give MitoSpinox **23**. Reduction of MitoSpinox **23** with glutathione or ascorbic acid is not surprising as the electron-withdrawing carbonyl group means that the O–H bond dissociation energy of the hydroxamic acid **22** will be at least 20 kJ mol^−1^ higher than those of simple hydroxylamines.^33^

The high propensity of MitoSpin **6** to be oxidized means that it will not be useful for distinguishing between different radicals in mitochondria. However, it is taken up by mitochondria and reacts with oxidizing agents to give a strong, simple EPR spectrum, so it may be useful for the detection of oxidative stress in mitochondria, when natural antioxidants are depleted. Indeed, its oxidation products could also be quantified by other techniques such as HPLC–MS as markers of oxidative stress. Furthermore, MitoSpin's ability to reduce oxidizing species, and its accumulation at the sites believed to be responsible for much of the endogenous oxidative stress in cells, gives it potential as a therapeutic antioxidant.^34^

In conclusion, we have demonstrated a novel Parham-type cyclization of an isocyanate to access an isoindolone skeleton, and used this as the key step in a concise synthesis of a new spin trap, MitoSpin **6**. MitoSpin **6** is a potentially useful mitochondria-targeted spin trap and could also be used therapeutically or as a probe to investigate mitochondrial oxidative stress in various models.

## 3 Experimental

### 3.1 General

#### 3.1.1 Synthesis

Reactions under an inert atmosphere were carried out using oven-dried or flame-dried glassware. Solutions were added via syringe. THF was freshly distilled from sodium benzophenone. Dichloromethane was distilled from CaH_2_ prior to use. Reagents were obtained from commercial suppliers and used without further purification unless otherwise stated. ^1^H and ^13^C NMR spectra were obtained on a Bruker DPX/400 spectrometer operating at 400 and 100 MHz, respectively. ^1^H NMR spectra in CDCl_3_ are referenced to residual chloroform at 7.26 ppm and in CD_3_OD are referenced to residual methanol at 3.31 ppm. ^13^C NMR spectra in CDCl_3_ are referenced to CDCl_3_ at 77.16 ppm and in CD_3_OD are referenced to CD_3_OD at 49.00 ppm. DEPT was used to assign the signals in the ^13^C NMR spectra as C, CH, CH_2_, or CH_3_. All coupling constants are measured in Hz. Mass spectra (MS) were recorded on a Jeol JMS700 (MStation) spectrometer. Some IR spectra were obtained employing a Golden Gate™ attachment that uses a type IIa diamond as a single reflection element so that the IR spectrum of the compound (solid or liquid) could be directly detected (thin layer) without any sample preparation.

#### 3.1.2 [4-(3′,3′-Dibutyl-2′-oxy-3′*H*-isoindol-5′-yloxy)butyl]triphenylphosphonium bromide (MitoSpin) **6**

A stirred solution of nitrone **19** (500 mg, 1.9 mmol), cesium carbonate (686 mg, 2.10 mmol), and 4-bromobutyltriphenylphosphonium bromide (1.83 g, 3.83 mmol) in dry MeOH (15 mL) were heated to reflux for 48 h. The mixture was then partitioned between H_2_O and EtOAc, and the aqueous phase was extracted with EtOAc (2×). The combined organic extracts were dried over MgSO_4_ and concentrated in vacuo to give a yellow solid foam. Recrystallization from EtOAc/hexane gave MitoSpin **6** as an amorphous solid (298 mg, 24%). Mp: decomposes at 84 °C. *δ*_H_ (CDCl_3_, 400 MHz): 7.92–7.87 (6H, m), 7.80–7.77 (3H, m), 7.70–7.66 (7H, m), 7.22 (1H, d, *J*=8.4 Hz), 6.85 (1H, dd, *J*=8.4, 2.0 Hz), 6.74 (1H, d, *J*=2.0 Hz), 4.18 (2H, t, *J*=5.8 Hz), 4.06–4.03 (2H, m), 2.35–2.25 (2H, m), 2.13–2.05 (2H, m), 1.96–1.63 (4H, m), 1.24–1.13 (4H, m), 0.99–0.82 (2H, m), 0.75 (6H, t, *J*=6.4 Hz), 0.61–0.48 (2H, m). *δ*_C_ (CDCl_3_, 100 MHz): 159.30 (C), 144.48 (C), 135.10 (CH), 133.82 (d, *J*=9.9 Hz, CH), 133.58 (CH), 130.55 (d, *J*=12.6 Hz, CH), 126.82 (C), 120.89 (CH), 118.84 (d, *J*=85.8 Hz, C) 114.24 (CH), 108.41 (CH), 84.18 (C), 67.19 (CH_2_), 37.21 (CH_2_), 29.62 (d, *J*=16.6 Hz, CH_2_), 24.83 (CH_2_), 22.51 (CH_2_), 22.05 (CH_2_), 19.52 (CH_2_), 13.90 (CH_3_). IR (KBr, cm^−1^): 3051, 2929–2858, 1683, 1611, 1586, 1526. LRMS (FAB/NOBA): 578 [M^+^ (phosphonium cation), 100]. HRMS: 578.3201. C_38_H_45_NO_2_P requires 578.3188.

#### 3.1.3 (2′-Bromo-5′-methoxyphenyl)acetic acid **9**

Bromine (8.3 mL, 162 mmol) was added dropwise to a solution of 3-methoxyphenylacetic acid **8** (25.7 g, 154 mmol) in CHCl_3_ (100 mL). The resulting mixture was allowed to stir overnight at rt and then poured into 20% aqueous sodium thiosulfate solution (300 mL). The layers were separated and the aqueous layer was extracted with CHCl_3_ (200 mL). The combined organics were washed with 20% aqueous sodium thiosulfate solution, dried over MgSO_4_ and concentrated in vacuo to give the aryl bromide **7** as an off-white solid (37.9 g, 100%); a small portion was recrystallized from EtOAc/hexane to give plates. Mp 108–109 °C. *δ*_H_ (CDCl_3_, 400 MHz): 7.46 (1H, d, *J*=8.8 Hz), 6.85 (1H, d, *J*=3.0 Hz), 6.74 (1H, dd, *J*=8.8, 3.0 Hz), 3.80 (2H, s), 3.78 (3H, s). *δ*_C_ (CDCl_3_, 100 MHz): 176.50 (C), 158.95 (C), 134.27 (C), 133.40 (CH), 117.29 (CH), 115.46 (C), 114.84 (CH), 55.51 (CH_3_), 41.49 (CH_2_). IR (KBr cm^−1^): 2949–2856, 1692, 1594, 1571. LRMS (EI^+^), *m*/*z*: 246 [M^+^^•^ (^81^Br), 28%)], 244 [M^+^^•^ (^79^Br), 28%], 201 (20), 199 (20), 165 (100). HMRS: 243.9734. C_9_H_9_BrO_3_ requires (M^+^^•^) 243.9735. ^1^H and ^13^C NMR matches literature.^35^

#### 3.1.4 Methyl 2-(2′-bromo-5′-methoxyphenyl)acetate **10**

Concentrated sulfuric acid (6 drops) was added to a solution of the carboxylic acid **9** (37.9 g, 155 mmol) in methanol (150 mL). The resulting mixture was stirred at rt overnight. The reaction was concentrated in vacuo to approximately 20 mL and the mixture taken up in EtOAc and washed with 1 M NaOH_(aq)_, then with brine. The organics were dried over MgSO_4_ and concentrated in vacuo to give ester **10** (37.6 g, 98%) as a yellow oil, which crystallized on standing to give prisms. Mp: 38–40 °C (lit.^36^ oil). *δ*_H_ (CDCl_3_, 400 MHz): 7.43 (1H, d, *J*=8.8 Hz), 6.84 (1H, d, *J*=3.0 Hz), 6.70 (1H, dd, *J*=8.8, 3.0 Hz), 3.77 (3H, s), 3.74 (2H, s), 3.71 (3H, s). *δ*_C_ (CDCl_3_, 100 MHz): 170.98 (C), 158.99 (C), 135.04 (C), 133.38 (CH), 117.22 (CH), 115.44 (C), 114.64 (CH), 55.53 (CH_3_), 52.29 (CH_3_), 41.73 (CH_2_). IR (thin layer, cm^−1^) 3007, 2952, 1743. LRMS (EI^+^), *m*/*z*: 260 [M^+^^•^ (^81^Br), 16%], 258 [M^+^^•^ (^79^Br), 16], 179 (100). HMRS: 257.9892 and 259.9871. C_10_H_11_^79^BrO_3_ requires (M^+^^•^) 257.9892, and C_10_H_11_^81^BrO_3_ requires (M^+^^•^) 259.9872. IR matches literature and ^1^H NMR spectrum is similar, but not the same as that recorded in a different solvent (CCl_4_).^36^

#### 3.1.5 Methyl 2-(2′-bromo-5′-methoxyphenyl)hexanoate **11**

*n*-BuLi (43 mL, 2.5 M in hexane, 109 mmol) was added dropwise to a stirred solution of diisopropylamine (15 mL, 109 mmol) in dry THF (150 mL) at −78 °C under argon ensuring that the internal temperature remained below −60 °C. The resulting solution was allowed to stir for 15 min and a solution of methyl ester **10** (25 g, 99 mmol) in dry THF (200 mL) was added dropwise ensuring that the internal temperature remained below −60 °C. The reaction mixture was allowed to stir for 15 min and then butyl iodide (12.0 mL, 109 mmol) was added dropwise. The mixture was allowed to warm to rt and stirred overnight. The reaction was quenched by the addition of ice cold H_2_O and extracted with Et_2_O. The organic layer was washed with H_2_O, dried over MgSO_4_, and concentrated in vacuo to give a brown oil. The oil was purified using flash chromatography on silica gel eluting with hexane followed by hexane/Et_2_O (3:2) to afford the ester **11** as a yellow oil (20.7 g, 91%). *R_f_* [SiO_2_, hexane/Et_2_O (3:2)]: 0.63. *δ*_H_ (CDCl_3_, 400 MHz): 7.43 (1H, d, *J*=8.8 Hz), 6.93 (1H, d, *J*=3.1 Hz), 6.67 (1H, dd, *J*=8.8, 3.1 Hz), 4.09 (1H, t, *J*=7.5 Hz), 3.77 (3H, s), 3.65 (3H, s), 2.07–1.98 (1H, m), 1.78–1.70 (1H, m), 1.36–1.21 (4H, m), 0.82 (3H, t, *J*=6.9 Hz). *δ*_C_ (CDCl_3_, 100 MHz): 174.04 (C), 159.21 (C), 139.87 (C), 133.46 (CH), 115.28 (CH), 114.34 (C), 114.34 (CH), 55.50 (CH_3_), 52.13 (CH_3_), 49.99 (CH), 33.16 (CH_2_), 29.62 (CH_2_), 22.58 (CH_2_), 13.98 (CH_3_). IR (thin layer, cm^−1^) 3010–2800, 1737, 1595, 1572. LRMS (EI^+^), *m*/*z*: 316 [M^+^^•^ (^81^Br), 49%], 314 [M^+^^•^ (^79^Br), 49%], 257 (12), 314 (12), 235 (100). HMRS: 314.0518 and 316.047. C_14_H_19_^79^BrO_3_ requires (M^+^^•^) 314.0518, and C_14_H_19_^81^BrO_3_ requires (M^+^^•^) 316.0499.

#### 3.1.6 Methyl 2-(2′-bromo-5′-methoxyphenyl)-2-butylhexanoate **12**

*n*-BuLi (39 mL, 2.5 M in hexane, 99 mmol) was added dropwise to a stirred solution of diisopropylamine (14 mL, 99 mmol) in dry THF (150 mL) at −78 °C under argon ensuring that the internal temperature remained below −60 °C. The resulting solution was allowed to stir for 15 min and a solution of ester **11** (28.4 g, 90.1 mmol) in dry THF (200 mL) was added dropwise ensuring that the internal temperature remained below −60 °C. The reaction mixture was allowed to stir for 15 min and then butyl iodide (11 mL, 99 mmol) was added dropwise. The mixture was allowed to warm to rt and stirred overnight. The reaction was quenched by the addition of ice cold H_2_O and extracted with Et_2_O. The organic layer was washed with H_2_O, dried over MgSO_4_, and concentrated in vacuo to give a brown oil. Flash chromatography on silica gel eluting with hexane followed by hexane/Et_2_O (3:2) afforded the ester **12** as a yellow oil, which crystallized on standing and was washed with hexane to give the ester as a an amorphous solid (22.9 g, 93%). Mp: 53–54 °C. *R_f_* [SiO_2_, hexane/Et_2_O (3:2)]: 0.65. *δ*_H_ (CDCl_3_, 400 MHz): 7.45 (1H, d, *J*=8.7 Hz), 6.91 (1H, d, *J*=3.0 Hz), 6.65 (1H, dd, *J*=8.7, 3.0 Hz), 3.81 (3H, s), 3.67 (3H, s), 2.20–2.12 (2H, m), 1.99–1.92 (2H, m), 1.32–1.24 (4H, m), 1.11–1.05 (2H, m), 0.96–0.90 (8H, m). *δ*_C_ (CDCl_3_, 100 MHz): 175.94 (C), 158.36 (C), 142.97 (C), 134.90 (CH), 116.87 (CH), 114.33 (C), 111.94 (CH), 55.41 (CH_3_), 54.48 (C), 52.15 (CH_3_), 33.26 (CH_2_), 26.15 (CH_2_), 23.11 (CH_2_), 14.01 (CH_3_). IR (thin layer, cm^−1^) 3010–2800, 1732, 1596. LRMS (EI^+^), *m*/*z*: 372 [M^+^^•^ (^81^Br), 3%], 370 [M^+^^•^ (^79^Br), 3], 291 (100). HRMS: 372.1125 and 370.1143. C_18_H_27_^81^BrO_3_ requires (M^+^^•^) 372.1125 and C_18_H_27_^79^BrO_3_ requires (M^+^^•^) 370.1144.

#### 3.1.7 2-(2′-Bromo-5′-methoxyphenyl)-2-butylhexanoic acid **13**

5 M KOH_(aq)_ (25 mL) was added to a stirred solution of ester **12** (10.0 g, 27.0 mmol) in DMSO (150 mL) and the resulting mixture was heated to reflux overnight. The reaction was allowed to cool, was acidified with 5 M HCl_(aq)_, and extracted with EtOAc (3×). The combined organic extracts were washed with brine, dried over MgSO_4_, and concentrated in vacuo to afford the carboxylic acid **13** as an off-white solid (8.08 g, 84%). A small portion was recrystallized from DCM to give needles. Mp: 163–165 °C. *δ*_H_ (CDCl_3_, 400 MHz): 7.46 (1H, d, *J*=8.6 Hz, H-3′), 6.91 (1H, d, *J*=3.0 Hz, H-6′), 6.66 (1H, dd, *J*=8.6 Hz, 3.0 Hz, H-4′), 3.80 (3H, s, CH_3_O), 2.24–2.16 (2H, m, CH_2_), 1.99–1.92 (2H, m, CH_2_), 1.33–1.11 (6H, m, 3×CH_2_), 0.99–0.92 (2H, m, CH_2_), 0.88 (6H, t, *J*=7.2 Hz, 2×CH_3_). *δ*_C_ (CDCl_3_, 100 MHz): 181.71 (C), 158.39 (C), 142.34 (C), 135.02 (CH), 116.99 (CH), 114.48 (C), 112.18 (CH), 55.46 (CH_3_), 54.54 (C), 32.94 (CH_2_), 26.09 (CH_2_), 23.12 (CH_2_), 14.03 (CH_3_). IR (KBr, cm^−1^): 3010–2955, 2610, 1703, 1597, 1570. LRMS (EI^+^), *m*/*z*: 358 [M^+^^•^ (^81^Br), 2%], 370 [M^+^^•^ (^79^Br), 2], 291 (M^+^^•^–^•^Br, 100), 85 (35). HRMS: 356.0986 and 358.0977. C_17_H_25_^79^BrO_3_ requires (M^+^^•^) 356.0987 and C_17_H_25_^81^BrO_3_ requires (M^+^^•^) 358.0969.

#### 3.1.8 1-Bromo-2-(5′-isocyanatonon-5′-yl)-4-methoxybenzene **14**

Triethylamine (9.2 mL, 66 mmol) was added to a stirred solution of carboxylic acid **13** (21.5 g, 60.2 mmol) in dry toluene (250 mL) at 0 °C under argon. Diphenylphosphoryl azide (14.3 mL, 66 mmol) was then added and the resulting mixture was allowed to stir for 15 min before heating to reflux for 18 h. The reaction was then quenched with saturated ammonium chloride solution and the mixture was extracted with Et_2_O (3×). The combined organics were washed with brine, dried over MgSO_4_, and concentrated in vacuo to giva a brown oil. Flash chromatography on silica gel eluting with hexane/EtOAc (4:1) gave the isocyanate **14** as an oil (20.8 g, 97%). *R_f_* [SiO_2_, hexane/Et_2_O (4:1)]: 0.78 *δ*_H_ (CDCl_3_, 400 MHz): 7.47 (1H, d, *J*=8.8 Hz), 7.25 (1H, d, *J*=2.8 Hz), 6.67 (1H, dd, *J*=8.8, 2.8 Hz), 3.82 (3H, s), 2.74–2.66 (2H, m), 1.90–1.83 (2H, m), 1.43–1.22 (6H, m), 1.00–0.84 (2H, m), 0.70 (6H, t, *J*=7.6 Hz). *δ*_C_ (CDCl_3_, 100 MHz): 158.78 (C), 141.40 (C), 136.22 (CH), 121.87 (C), 117.41 (CH), 113.62 (CH), 109.49 (C), 70.77 (C), 55.55 (CH_3_), 40.84 (CH_2_), 26.59 (CH_2_), 22.73 (CH_2_), 14.07 (CH_3_). IR (Thin layer, cm^−1^) 3010–2850, 2261, 1592, 1569. MS, (EI^+^), *m*/*z*: 355 [M^+^^•^ (^81^Br), 36%], 353 [M^+^^•^ (^79^Br), 36], 298 (100), 296 (100), 242 (60), 240 (61), 201 (39), 199 (36), 174 (81). HMRS: 353.0991 and 355.0968. C_17_H_24_^79^BrNO_2_ requires (M^+^^•^) 353.0990 and C_17_H_24_^81^BrNO_2_ requires (M^+^^•^) 355.0970.

#### 3.1.9 3,3-Dibutyl-2,3-dihydro-5-methoxyisoindol-1-one **15**

^*t*^BuLi (24 mL, 1 M in cyclohexane, titrated using 1,3-diphenyl acetone-*p*-tosylhydrazone, 24 mmol) was added dropwise to a stirred solution of the isocyanate **14** (4.00 g, 11.8 mmol) in dry THF (100 mL) at −78 °C under argon. The resulting mixture was allowed to warm to rt and stirred overnight before ice cold water was added. The mixture was extracted with EtOAc and the organic layer was washed with brine, dried over MgSO_4_, and concentrated in vacuo to afford amide **15** as an amorphous solid (3.02 g, 93%). Mp: 96–97 °C. *δ*_H_, (CDCl_3_, 400 MHz): 7.71 (1H, d, *J*=8.4 Hz), 6.93 (1H, dd, *J*=8.4, 2.2 Hz), 6.87 (1H, s), 6.75 (1H, d, *J*=2.2 Hz), 3.87 (3H, s), 1.88–1.72 (4H, m), 1.24–1.14 (6H, m), 0.84–0.76 (8H, m). *δ*_C_ (CDCl_3_, 100 MHz): 170.56 (C), 163.16 (C), 153.23 (C), 125.17 (CH), 125.06 (C), 113.85 (CH), 106.50 (CH), 64.80 (C), 55.75 (CH_3_), 39.24 (CH_2_), 25.62 (CH_2_), 22.90 (CH_2_), 14.01 (CH_3_). IR (KBr, cm^−1^) 3286, 2955–2850, 1693, 1661, 1606, 1488. LRMS (EI^+^), *m*/*z*: 275 (M^+^^•^, 1.5%), 218 (100). HRMS: 275.1884. C_17_H_25_O_2_N requires (M^+^^•^) 275.1885.

#### 3.1.10 1,1-Dibutyl-2,3-dihydro-6-methoxy-1*H*-isoindole **16**

Borane/THF complex (51 mL of a 1 M solution in THF, 51 mmol) was added over 20 min to a stirred solution of amide **15** (3.50 g, 12.7 mmol) in dry THF (50 mL) at 0 °C under argon and the mixture allowed to stir for 1 h. After this time the mixture was heated to reflux for 48 h. The reaction was then quenched by the slow addition of ice cold H_2_O (100 mL). 1 M NaOH_(aq)_ was added and the mixture extracted with Et_2_O (2×). The combined organic extracts were washed with brine (100 mL), dried over MgSO_4_, and then concentrated in vacuo to give an oil **16** (3.30 g, 99%). *δ*_H_ (CDCl_3_, 400 MHz): 7.16 (1H, d, *J*=8.4 Hz), 6.77 (1H, dd, *J*=8.4, 2.4 Hz), 6.61 (1H, d, *J*=2.4 Hz), 4.14 (2H, s), 3.82 (3H, s), 2.05–1.65 (4H, m), 1.26–1.23 (6H, m), 1.10–1.04 (2H, m), 0.89 (6H, t, *J*=7.2 Hz). The amine was dissolved in Et_2_O (20 mL) and ethereal HCl was added affording the salt as a white precipitate. Mp: decomposed at 210 °C. *δ*_H_ (CDCl_3_, 100 MHz): 10.19 (2H, s), 7.17 (1H, d, *J*=8.4 Hz), 6.88 (1H, dd, *J*=8.4, 2.2 Hz), 6.59 (1H, d, *J*=2.2 Hz), 4.60 (2H, s), 3.82 (3H, s), 2.13–1.96 (4H, m), 1.57–1.48 (2H, m), 1.42–1.23 (6H, m), 0.91 (6H, t, *J*=7.2 Hz). *δ*_C_ (CDCl_3_, 100 MHz): 160.37 (C), 143.48 (C), 125.85 (C), 124.00 (CH), 114.45 (CH), 108.28 (CH), 75.76 (C), 55.76 (CH_3_), 48.62 (CH_2_), 37.40 (CH_2_), 25.73 (CH_2_), 22.82 (CH_2_), 14.05 (CH_3_). IR (KBr, cm^−1^): 3010–2800, 2717, 1621, 1588. MS, (CI^+^), *m*/*z*: 262 [M+H^+^ (amine), 82%], 218 (24), 79 (100). HRMS: 262.2172. C_17_H_28_ON requires [M+H^+^ (amine)] 262.2171.

#### 3.1.11 1,1-Dibutyl-6-methoxy-1*H*-isoindole-2-oxide **17**

The hydrochloride salt of amine **16** (500 mg, 1.68 mmol) was dissolved in MeOH (5 mL) then NaOH (70.5 mg, 1.68 mmol), Na_2_WO_4_ (58.0 mg, 0.17 mmol), and 30% H_2_O_2_ in H_2_O (0.57 mL, 5.04 mmol) were added. The resulting mixture was then allowed to stir at rt under argon for 3 days. The reaction was then quenched with 20% Na_2_S_2_O_3_ in brine and extracted into EtOAc (3×). The organic extracts were combined, dried over MgSO_4_, and concentrated to give an orange oil. Column chromatography, on silica gel, eluting with EtOAc/hexane (3:7) gave the nitrone **17** as a pale orange solid (157 mg, 34%). *δ*_H_ (CDCl_3_, 400 MHz): 7.67 (1H, s), 7.25 (1H, d, *J*=8.4 Hz), 6.88 (1H, dd, *J*=8.4, 1.6 Hz), 6.75 (1H, d, *J*=1.6 Hz), 3.87 (3H, s), 2.14–2.07 (2H, m), 1.87–1.79 (2H, m), 1.30–1.11 (4H, m), 1.02–0.91 (2H, m, CH_2_), 0.77 (6H, t, *J*=7.2 Hz), 0.64–0.58 (2H, m). *δ*_C_ (CDCl_3_, 100 MHz): 160.09 (C), 144.51 (C), 133.69 (C), 126.98 (C), 120.95 (CH), 113.01 (CH), 108.41 (CH), 84.23 (C), 55.75 (CH_3_), 37.33 (CH_2_), 24.90 (CH_2_), 22.61 (CH_2_), 13.95 (CH_3_). IR (KBr, cm^−1^): 3100–2800, 1588, 1525. LRMS (EI^+^), *m*/*z*: 275 (M^+^^•^, 18%), 258 (82), 219 (100), 202 (41), 176 (96), 160 (32). HRMS: 275.1887. C_17_H_25_O_2_N requires (M^+^^•^) 275.1885.

#### 3.1.12 3,3-Dibutyl-2,3-dihydro-1*H*-isoindol-5-ol **18**

BBr_3_ (54 mL of a 1 M solution in DCM, 54 mmol) was added dropwise to a solution of amine **16** (4.00 g, 15.3 mmol) in dry DCM (100 mL) at 0 °C under argon. The reaction mixture was then allowed to warm to rt for 72 h. After this time the reaction was quenched with ice cold water. The mixture was basified with 1 M NaOH_(aq)_ and extracted with DCM (3×). The combined organics were washed with brine, dried over MgSO_4_, and concentrated in vacuo to give phenol **18** as an amorphous solid (3.52 g, 93%). Mp: 110–111 °C. *δ*_H_ (CD_3_OD, 400 MHz): 7.21 (1H, d, *J*=8.3 Hz), 6.84 (1H, dd, *J*=8.3, 2.2 Hz), 6.65 (1H, d, *J*=2.2 Hz), 4.46 (2H, s), 2.09–2.02 (2H, m), 1.93–1.85 (2H, m), 1.45–1.32 (6H, m), 1.26–1.16 (2H, m), 0.88 (6H, t, *J*=7.1 Hz). *δ*_C_ (CD_3_OD, 100 MHz): 159.74 (C), 143.85 (C), 125.14 (C), 125.04 (CH), 117.61 (CH), 110.34 (CH), 76.59 (C), 49.65 (CH_2_), 37.42 (CH_2_), 26.53 (CH_2_), 23.79 (CH_2_), 14.17 (CH_3_). IR (KBr, cm^−1^): 3433, 3310, 2955–2931, 2858, 1612, 1481, 1465. LRMS (CI^+^), *m*/*z*: 248 [M+H^+^ (amine), 100%], 190 (25). HRMS: 248.2010. C_16_H_26_NO requires [M+H^+^ (amine)] 248.2015.

#### 3.1.13 3,3-Dibutyl-2-oxy-3*H*-isoindol-5-ol **19**

Sodium tungstate (1.00 g, 3.0 mmol) and hydrogen peroxide (2.1 mL, 90 mmol) were added to a stirred solution of amine **18** (1.50 g, 6.1 mmol) in dry methanol (20 mL) and the resulting mixture was stirred at rt for 72 h. The reaction mixture was then quenched with 20% aqueous sodium sulfite solution and extracted with DCM (3×). The combined organic extracts were washed with brine, dried over MgSO_4_, and concentrated in vacuo to give an orange foam. The foam was triturated with EtOAc to give phenol **19** as an off-white solid (980 mg, 61%). A small portion was recrystallized from hexane/EtOAc to give prisms. Mp: 193–195 °C. *δ*_H_ (CD_3_OD, 400 MHz): 8.12 (1H, s), 7.32 (1H, d, *J*=8.4 Hz), 6.81 (1H, dd, *J*=8.4, 2.0 Hz), 6.74 (1H, d, *J*=2.0 Hz), 2.14–2.01 (2H, m), 1.94–1.78 (2H, m), 1.23–1.06 (4H, m), 0.95–0.85 (2H, m), 0.80 (6H, t, *J*=6.4 Hz), 0.74–0.70 (2H, m, CH_2_). *δ*_C_ (CD_3_OD, 100 MHz): 160.82 (C), 146.81 (C), 139.29 (CH), 125.71 (C), 123.72 (CH), 116.73 (CH), 110.07 (CH), 85.11 (C), 37.69 (CH_2_), 25.88 (CH_2_), 23.38 (CH_2_), 14.14 (CH_3_). IR (KBr, cm^−1^): 3439 (OH), 2954–2857, 1587, 1537. LRMS (EI^+^), *m*/*z*: 261 (M^+^^•^, 10%), 244 (M^+^^•^−^•^OH, 23), 216 (M^+^^•^−H_2_O and HCN, 58), 205 (M^+^^•^−C_4_H_8_, 69%), 188 (M^+^^•^−^•^OH and C_4_H_8_, 37), 162 (M^+^^•^−C_4_H_8_ and ^•^Pr, 100), 146 (M^+^^•^−^•^Bu and BuH, 32). HRMS: 261.1728. C_16_H_23_NO_2_ requires (M^+^^•^) 261.1729.

#### 3.1.14 EPR spectroscopy

EPR spectra were acquired on a bench-top EPR machine with a permanent magnet and a magnetic sweep circuit (center of field=0.345 T, sweep width 25 mT) operating at a frequency of 9.8 GHz (X-band). Acquisition parameters: RG 3.99×10^3^, 2.76 mW, MA 0.5 G. Hyperfine couplings were derived from simulations and the *g* value calculated using strong pitch as the reference.

#### 3.1.15 Reaction between MitoSpin 4 and hydroxy radicals in the presence of iron ions

Aqueous iron(II) sulfate (100 μL of a 1 mM solution) and aqueous hydrogen peroxide (100 μL of a 1 mM solution) were added to a solution of the MitoSpin **4** in DMF (100 μL of a 10 mM solution). The solution was then immediately transferred to a quartz flat cell and placed in the EPR spectrometer for analysis.

#### 3.1.16 Reaction between MitoSpin 4 and hydroxyl radicals generated by UV irradiation

Aqueous hydrogen peroxide (150 μL of a 100 μM solution) was added to a solution of MitoSpin **4** in DMF (150 μL of a 30 mM solution) in a quartz cuvette and the solution was irradiated with UV light (254 nm, 12 W lamp) for 20 min before transferring to a quartz flat cell and placed in the EPR spectrometer for analysis.

#### 3.1.17 Mitochondrial incubations

Rat liver mitochondria were prepared by homogenization followed by differential centrifugation in 250 mM sucrose, 5 mM Tris–HCl, 1 mM EGTA, pH 7.4.^37^ Protein concentration was determined by the biuret assay using BSA as a standard.^38^ All incubations were at 30 °C in KCl buffer (120 mM KCl, 1 mM EGTA, 10 mM HEPES, pH 7.2) supplemented with 10 mM succinate and 4 μg/mL rotenone. A TPP-selective electrode was constructed and used as previously described^26^ to measure MitoSpin accumulation into rat liver mitochondria.

## References

[1] Wallace D.C. (1999). Science.

[2] Finkel T. (2005). Nat. Rev. Mol. Cell Biol..

[3] Murphy M.P. (2009). Biochem. J..

[4] Rosen G.M., Britigan B.E., Halpern H.J., Pou S. (1999). Free Radicals: Biology and Detection by Spin Trapping.

[5] Murphy M.P., Echtay K.S., Blaikie F.H., Asin-Cayuela J., Cocheme H.M., Green K., Buckingham J.A., Taylor E.R., Hurrell F., Hughes G., Miwa S., Cooper C.E., Svistunenko D.A., Smith R.A., Brand M.D. (2003). J. Biol. Chem..

[6] Hardy M., Chalier F., Ouari O., Finet J.P., Rockenbauer A., Kalyanaraman B., Tordo P. (2007). Chem. Commun..

[7] Hardy M., Rockenbauer A., Vasquez-Vivar J., Felix C., Lopez M., Srinivasan S., Avadhani N., Tordo P., Kalyanaraman B. (2007). Chem. Res. Toxicol..

[8] Xu Y., Kalyanaraman B. (2007). Free Radical Res..

[9] Ross M.F., Kelso G.F., Blaikie F.H., James A.M., Cocheme H.M., Filipovska A., Da Ros T., Hurd T.R., Smith R.A., Murphy M.P. (2005). Biochemistry (Mosc).

[10] Murphy M.P., Smith R.A. (2007). Annu. Rev. Pharmacol. Toxicol..

[11] Murphy M.P., Smith R.A.J. (2000). Adv. Drug Delivery Rev..

[12] Smith R.A.J., Porteous C.M., Coulter C.V., Murphy M.P. (1999). Eur. J. Biochem..

[13] Smith R.A.J., Porteous C.M., Gane A.M., Murphy M.P. (2003). Proc. Natl. Acad. Sci. U.S.A..

[14] Burns R.J., Smith R.A.J., Murphy M.P. (1995). Arch. Biochem. Biophys..

[15] Lin T.K., Hughes G., Muratovska A., Blaikie F.H., Brookes P.S., Darley-Usmar V., Smith R.A.J., Murphy M.P. (2002). J. Biol. Chem..

[16] Robinson K.M., Janes M.S., Pehar M., Monette J.S., Ross M.F., Hagen T.M., Murphy M.P., Beckman J.S. (2006). Proc. Natl. Acad. Sci. U.S.A..

[17] Trnka J., Blaikie F.H., Smith R.A.J., Murphy M.P. (2008). Free Radical Biol. Med..

[18] Marx L., Chiarelli R., Guiberteau T., Rassat A. (2000). J. Chem. Soc., Perkin Trans. 1.

[19] Blinco J.P., Hodgson J.L., Morrow B.J., Walker J.R., Will G.D., Coote M.L., Bottle S.E. (2008). J. Org. Chem..

[20] Cooper A. (2004). Biophysical Chemistry.

[21] Fevig T.L., Bowmen S.M., Janowick D.A., Jones B.K., Munson H.R., Ohlweiler D.F., Thomas C.E. (1996). J. Med. Chem..

[22] Orito K., Horibata A., Nakamura T., Ushito H., Nagasaki H., Yaguchi M., Yamashita S., Tokuda M. (2004). J. Am. Chem. Soc..

[23] Parham W.E., Bradsher C.K. (1982). Acc. Chem. Res..

[24] Couture A., Deniau E., Lamblin M., Lorion M., Grandclaudon P. (2007). Synthesis.

[25] Kuramochi K., Watanabe H., Kitahara T. (2000). Synlett.

[26] Asin-Cayuela J., Manas A.R., James A.M., Smith R.A., Murphy M.P. (2004). FEBS Lett..

[27] Smith R.A., Kelso G.F., James A.M., Murphy M.P. (2004). Methods Enzymol..

[28] Ross M.F., Da Ros T., Blaikie F.H., Prime T.A., Porteous C.M., Severina I.I., Skulachev V.P., Kjaergaard H.G., Smith R.A., Murphy M.P. (2006). Biochem. J..

[29] Ross M.F., Prime T.A., Abakumova I., James A.M., Porteous C.M., Smith R.A.J., Murphy M.P. (2008). Biochem. J..

[30] Rosen G.M., Rauckman E.J. (1980). Mol. Pharmacol..

[31] Saldyka M., Mielke Z. (2007). Vib. Spectrosc..

[32] Makino K., Hagi A., Ide H., Murakami A. (1992). Can. J. Chem..

[33] Luo Y.-R. (2003). Handbook of Bond Dissociation Energies in Organic Compounds.

[34] Floyd R.A., Kopke R.D., Choi C.-H., Foster S.B., Doblas S., Towner R.A. (2008). Free Radical Biol. Med..

[35] Jeong J.H., Weinreb S.M. (2006). Org. Lett..

[36] Ambros R., von Angerer S., Wiegrebe W. (1988). Arch. Pharm. (Weinheim).

[37] Chappell J.B., Hansford R.G., Birnie G.D. (1972). Subcellular Components: Preparation and Fractionation.

[38] Gornall A.G., Bardawill C.J., David M.M. (1949). J. Biol. Chem..

